# Serum Urate and Atrial Fibrillation: A Bidirectional Mendelian Randomization Study

**DOI:** 10.1002/clc.70089

**Published:** 2025-01-27

**Authors:** Shandong Yu, Wei Wang, Yulian Gao, Jun Zhou, Yanpeng Chu

**Affiliations:** ^1^ Department of Cardiology Capital Medical University Affiliated Beijing Friendship Hospital Beijing China; ^2^ Department of Cardiology Dazhou Central Hospital Dazhou Sichuan Province China; ^3^ School of Clinical Medicine, North Sichuan Medical College Nanchong Sichuan Province China; ^4^ School of Clinical Medicine Southwest Medical University Luzhou Sichuan Province China; ^5^ Medical College Sichuan University of Arts and Sciences Dazhou Sichuan Province China

**Keywords:** atrial fibrillation, causal association, Mendelian randomization, risk factor, serum urate

## Abstract

**Background:**

Observational studies indicate that serum urate level is associated with atrial fibrillation (AF). However, whether this association is causal remains controversial, due to confounding factors and reverse causality. We aim to evaluate the causal relationship of genetically predicted serum urate level with AF.

**Methods:**

A bidirectional Mendelian randomization (MR) study was performed. Instrumental variables were obtained from the Global Urate Genetics Consortium (110347 individuals). We obtained summary statistics of AF from two genome‐wide association studies (GWAS) data sets for AF. Inverse‐variance‐weighted method was applied to obtain MR estimates and other statistical methods were conducted in the sensitivity analyses. The reverse MR analysis was performed to evaluate the effect of AF on serum urate levels.

**Results:**

Genetically determined serum urate level was not associated with AF in two studies (OR, 1.03; 95% CI, 0.95–1.11; *p* = 0.47); (OR, 1.06; 95% CI, 0.99–1.12; *p* = 0.09). The main results kept robust in the most sensitivity analyses. Multivariable MR analyses suggested that the association pattern did not change, after adjusting for body mass index (BMI), HbA1c: hemoglobin A1c (HbA1c), hypertension, low‐density lipoprotein cholesterol (LDL‐C) and coronary heart disease. No causal effect of AF on serum urate levels was found in two studies (OR, 1.02; 95% CI, 0.98–1.05; *p* = 0.30); (OR, 1.00; 95% CI, 0.98–1.03; *p* = 0.95).

**Conclusions:**

Our MR study supports no bidirectional causal effect of serum urate levels and AF. This implies that treatments aimed at lowering uric acid may not reduce the risk of AF.

## Graphical abstract

In this study, we employed Mendelian Randomization (MR) methods to assess the causal relationship between genetically predicted serum urate levels and atrial fibrillation (AF). Our findings indicate no causal association between genetically determined serum urate levels and AF, a pattern that remained consistent across two different AF GWAS data sets and did not change after adjusting for potential confounders in multivariable MR analyses.

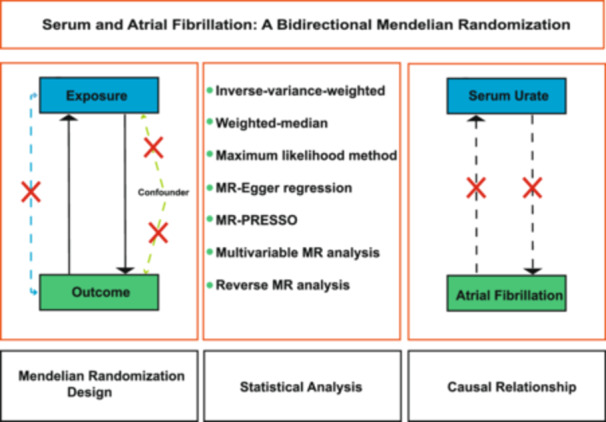

## 1 Introduction

Atrial fibrillation (AF) is the most prevalent tachycardia worldwide [1, 2]. It is a significant risk factor for ischemic stroke, heart failure, dementia, and cardiovascular motality [3]. The global trend of population aging suggests a predicted, continuous rise in the AF prevalence, accompanied by a growing socioeconomic burden [4].

Several studies have reported that oxidative stress is vital in AF development [5, 6, 7]. Uric acid (UA) promotes oxidative stress [8]. Elevated serum UA is related to various cardiovascular diseases (CVDs), including hypertension, heart failure, and coronary atherosclerotic heart disease [9]. The correlation between serum UA and AF has been found in several observational studies [10], albeit with controversial results [10]. There are several co‐morbid factors, all of which overlap and are correlated with one another. It is still uncertain whether the association between hyperuricemia and AF is merely a coincidental occurrence or a direct causative relationship. Confounding variables and the possibility of reverse causation contribute to the lack of clarity around this connection.

Mendelian randomization (MR) analysis, a method that employs instrumental variables (IV) derived from gene mutations with established functions, has been extensively utilized in assessing the potential causative influence of exposure on outcomes [11, 12]. The estimations are less susceptible to reverse causation, inaccuracies in measurement, and environmental confounders [13]. Therefore, genome‐wide association studies (GWASs) were employed to examine the genetically defined causality relationship between high serum urate levels and AF utilizing the bidirectional MR design. In addition, the primary analyses were repeatedly conducted using AF‐related data sets obtained from different sources to determine the degree to which the findings were consistent. At the same time, the causal impact of AF on serum urate level was examined.

## 2 Methods

### 2.1 Study Design

The cause‐effect correlation between serum urate and AF risk was assessed using a two‐sample bidirectional MR analysis (Supporting Information S1: Figure S1).

The MR analyses were completed in compliance with the Strengthening the Reporting of Observational Studies in Epidemiology‐MR (STROBE‐MR) guidelines [14].

Serum urate was considered the exposure, while AF was designated as the outcome. The three assumptions below underpin the use of IVs in assessing serum urate concentrations: (i) relevance assumption, that is, the genetic variations in question should robustly correlate with serum urate concentrations; (ii) independence assumption, that is, the genetic variations are not influenced by potential confounding variables, and (iii) exclusion restriction, that is, these genetic variants should exclusively link to AF risk due to alterations in serum urate levels [11]. Conversely, the potential for reverse causation was considered when evaluating the impact of AF on serum urate levels. For this reason, only single nucleotide polymorphisms (SNPs) linked to serum urate concentrations at genome‐wide significance were incorporated into the analyses, ensuring the strong relevance of all selected instruments to the exposure. Furthermore, it's essential that these genetic variations maintained their independence and didn't show linkage disequilibrium (LD), signifying a randomized distribution at the time of conception. In light of potential horizontal pleiotropy, further investigations using alternative statistical methods are needed.

### 2.2 Genetic Instrument Selection

Based on a meta‐analysis of 48 GWASs involving 110 347 Europeans by the Global Urate Genetics Consortium (GUGC) (weblink: https://gwas.mrcieu.ac.uk/, ID: ieu‐a‐1055), IVs pertaining to human serum urate concentrations were identified [15]. The average age was 52.12 years, and men constituted 45.15% of the population. The mean (standard deviation; SD) concentrations of urate in the serum varied between 3.86 (0.92) and 6.10 (1.46) mg/dL. Subsequently, the uricase approach was applied in the majority of the studies to determine urate levels.

A genome‐wide significance criterion of *p* < 5 × 10^−8^ was met for the identification of SNPs linked to serum urate levels. These SNPs were subjected to a test for LD to group them based on their degree of independence. There was a pruning of genetic variations in LD pairs with an *r* ^2^ > 0.001. The potency of IVs was evaluated using *R* ^2^ and F‐statistics in accordance with the sample size of the exposure data set, number of IVs, and genetic variation [16]. In cases of LD between SNPs, the variant with a higher *p* value was excluded. Eventually, the final IVs for serum urate concentration were constructed from 27 SNPs, as detailed in Supporting Information S1: Tables S1–S7.

### 2.3 Data Sources

Data on AF genetic associations at the summary level were sourced from two earlier multiethnic GWAS meta‐analyses [17, 18]. The research conducted by Roselli analyzed data from more than 588 190 individuals (of which 65 446 were AF cases), the majority of which were drawn from the UK Biobank, Broad AF, AFGen, and Biobank Japan investigations. Of these participants, the majority (84.2%) had European ancestry, followed by 12.5% with East Asian ancestry, 2.0% of African American descent, and 1.3% of Hispanic or Brazilian descent (weblink: https://www.ebi.ac.uk/gwas/, ID: GCST006061). Nielsen's study assessed > 1 000 000 individuals (60 620 AF cases) from six studies, including the AFGen Consortium, UK Biobank, DiscovEHR, deCODE, the Michigan Genomics Initiative (MGI), and the Nord‐Trøndelag Health Study (HUNT). This research identified 111 regions in the genome where at least one variation was correlated with a higher risk of AF (weblink: https://www.ebi.ac.uk/gwas/, ID: GCST006414) [18]. The summary‐level data for genetic correlations with AF are demonstrated in Supporting Information S1: Tables S2 and S3.

The study retrieved genetic associations from various consortiums (Date accessed: June 17–19, 2023). The associations for BMI were derived from the Genetic Investigation of Anthropometric Traits (GIANT) consortium (weblink: https://gwas.mrcieu.ac.uk/, ID: ieu‐a‐835) [19], hypertension from the International Consortium for Blood Pressure (ICBP) (weblink: https://gwas.mrcieu.ac.uk/, ID: ukb‐b‐14177) [20], hemoglobin A1c (HbA1c) from the Meta‐Analyses of Glucose and Insulin‐Related Traits Consortium (MAGIC) (weblink: https://gwas.mrcieu.ac.uk/, ID:ieu‐b‐103) [21], low‐density lipoprotein cholesterol (LDL‐C) from the Global Lipids Genetics Consortium (GLGC) (weblink: https://www.ebi.ac.uk/gwas/, ID: GCST002222) [21], and coronary heart disease from CARDIoGRAMplusC4D (weblink: https://www.ebi.ac.uk/gwas/, ID: GCST003116) [22]. Ethical review committees had approved all the studies included in the GWAS, with participants providing written informed consent. All data from this study are available for public access.

### 2.4 Statistical Analysis

The primary statistical model employed was the multiplicative random‐effects inverse‐variance‐weighted (IVW) method, which estimated the causal influence of serum urate concentrations on AF risk. Additionally, the robustness of relationships and the possible pleiotropy were evaluated by performing supplementary sensitivity analyses utilizing the MR‐Egger method, maximum likelihood, weighted‐median, and MR Pleiotropy Residual Sum and Outlier (MR‐PRESSO) methods [23, 24, 25]. The weighted‐median procedure is designed to offer reliable causal estimations even when up to half of the instrument weight is from invalid instruments [23]. Potential pleiotropy in the relationships could be determined by using intercept tests in MR‐Egger regression. Moreover, by adjusting for pleiotropy, the MR‐Egger regression offered a refined estimate. Nevertheless, this method may compromise the power of statistical analyses [24]. The MR‐PRESSO approach managed to pinpoint possible outliers and offer unbiased causal estimations after eliminating the detected outliers as well as the variance between estimates via distortion tests [25]. Genetic correlations between AF and serum urate concentrations were analyzed utilizing leave‐one‐out methods and visualized using scatter plots. The multivariable MR analysis enabled the assessment of the potential confounding factors, particularly HbA1c, hypertension, BMI, CHD, and LDL‐C, in affecting the relationships between genetically inferred serum urate and AF [26].

The effect of an SNP on the concentration of urate in the serum and AF should be attributed to the same allele. Any SNP for which the alleles failed to match were omitted. If a particular SNP was missing from the outcome data set, its presence can be verified using the SNiPa database (http://snipa.helmholtz-muenchen.de/snipa3/). The genotype data from the European population collected during Phase 3 of the 1000 Genomes Project has been employed by this online platform. Another SNP that was shown to be in LD with the specified SNP (*r* ^2^ > 0.8) would be designated as a suitable proxy for the analysis.

The power estimation was analyzed with the assistance of another web‐based platform, known as mRnd (https://shiny.cnsgenomics.com/mRnd/), to ensure that the minimal impact was more than 80% power [27]. The most important factors considered in this calculation included the sample size, the Type I error rate, the proportion of cases, the odds ratio (OR) related to the outcome, and the proportion of variance for the connection between SNPs and exposure variables (*r* ^2^). Then, the threshold for statistical significance was established at a two‐sided *p* value of 0.017 (*p* = 0.05/3 tests), considering the Bonferroni adjustment for multiple tests. The statistical tests and analyses for this investigation were carried out utilizing the R software (version 4.2.3; R Foundation for Statistical Computing, Vienna, Austria) and R package “TwoSampleMR” (https://github.com/MRCIEU/TwoSampleMR), along with “MR‐PRESSO” (https://github.com/rondolab/MR-PRESSO) [28].

## 3 Results

Twenty‐seven LD‐independent SNPs were determined as IVs for urate. These IVs all had F‐statistics greater than the threshold of 10. This finding demonstrated that the IVs were reliable instruments, lowering the bias associated with IV estimations (Supporting Information S1: Table S1) [29]. The MR findings revealed that genetically inferred serum urate level was unrelated to AF based on genetics (OR, 1.03; 95% confidence interval [CI], 0.95–1.11; *p* = 0.47] in the IVW model (Supporting Information S1: Table S4, Supporting Information S1: Figure S2). Weighted Median, MR‐Egger, and MR‐PRESSO all yielded findings that were in line with those of the IVW model. MR‐Egger regression depicted no directional pleiotropy (*p* for intercept = 0.11) (Supporting Information S1: Table S4). No evidence of possible directional pleiotropy in the MR‐PRESSO test was witnessed, with a stable estimate (OR, 1.03; 95% CI, 0.95–1.11; *p* = 0.47) after removing the three outliers (Supporting Information S1: Table S4).

The impacts of serum urate levels on AF were not mediated by any single SNP, as shown by a leave‐one‐out analysis (Supporting Information S1: Figure S3). Scatter plots visually represented the correlation between AF and serum urate concentration (Supporting Information S1: Figure S4). The funnel plot is shown in Supporting Information S1: Figure S5. Moreover, multivariable MR analyses demonstrated that the correlation pattern remained unchanged after controlling for hypertension, LDL‐C, CHD, HbA1c, or BMI (Table 1).

**Table 1:** Multivariable Mendelian randomization associations of serum urate with atrial fibrillation adjusting for cardiovascular risk factors in Roselli's study.

Model	Direct effect	OR	95% CI	*p* value
Adjusted for BMI	0.0042	1.00	0.95–1.07	0.89
Adjusted for LDL‐c	0.043	1.04	0.98–1.11	0.15
Adjusted for CHD	0.029	1.02	0.95–1.11	0.46
Adjusted for hypertension	0.024	1.02	0.95–1.11	0.54
Adjusted for HbA1C	0.036	1.04	0.96–1.12	0.34
Adjusted for all	0.0042	1.00	0.95–1.07	0.89

Abbreviations: BMI, body mass index; CHD, coronary heart disease; HbA1C, hemoglobin A1c; LDL‐c, low‐density lipid cholesterol.

To evaluate whether the link between serum urate concentration and AF was consistent across individuals from different data sources, the primary analyses were repeatedly conducted using genetic correlations from an additional GWAS [18]. The correlation of serum urate with AF remained in IVW model (OR, 1.06; 95% CI, 0.99–1.12; *p* = 0.09) (Supporting Information S1: Figure S6, Supporting Information S1: Table S5), Weighted Median (OR, 1.02; 95% CI, 0.97–1.08; *p* = 0.47), MR‐Egger (OR, 0.95; 95% CI, 0.85–1.05; *p* = 0.29) and MR‐PRESSO(OR, 1.05; 95% CI, 0.99–1.12; *p* = 0.10) models (Supporting Information S1: Table S5). However, when two outlier SNPs were removed, the effect of serum urate on AF revealed a marginal elevation in the MR‐PRESSO model (Supporting Information S1: Table S5). Using a leave‐one‐out approach, we found that no specific individual SNP had a substantial impact on the correlation between serum urate and AF risk (Supporting Information S1: Figure S7).

Scatter plots visually represented the association between serum urate levels and AF (Supporting Information S1: Figure S8). The funnel plot is shown in Supporting Information S1: Figure S9. Moreover, multivariable MR analyses depicted no association pattern alteration even after adjusting for variables like hypertension, LDL‐C, HbA1c, BMI, or CHD (Table 2).

**Table 2:** Multivariable Mendelian randomization associations of serum urate with atrial fibrillation adjusting for cardiovascular risk factors in Nielsen's study.

Model	Direct effect	OR	95% CI	*p* value
Adjusted for BMI	0.063	1.06	1.01–1.12	0.89
Adjusted for LDL‐c	0.042	1.04	0.99–1.10	0.14
Adjusted for CHD	0.046	1.05	0.98–1.12	0.16
Adjusted for hypertension	0.034	1.03	0.96–1.12	0.39
Adjusted for HbA1C	0.051	1.05	0.99–1.12	0.11
Adjusted for all	0.008	1.00	0.95–1.07	0.80

Abbreviations: BMI, body mass index; CHD, coronary heart disease; HbA1C, hemoglobin A1c; LDL‐c, low‐density lipid cholesterol.

Further, we completed a reverse‐direction MR analysis to examine the potential reverse causality. Roselli's and Nielsen's studies used 77 and 83 SNPs as the IVs for AF, respectively. The combined analysis of all the individual SNPs provided no substantial evidence supporting a causal relationship between AF and serum urate level (Supporting Information S1: Tables S6 and S7, Supporting Information S1: Figures S10, S12, S14, S16).

Additionally, both sensitivity and leave‐one‐out analyses confirmed the consistency and stability of the underlying relationship pattern (Supporting Information S1: Figures S11, S13, S15, S17). After adjusting for hypertension, LDL‐C, HbA1c, BMI, or CHD, the multivariate MR analysis found no significant alteration in the relationship pattern (Tables 3 and 4).

**Table 3:** Multivariable Mendelian randomization associations of atrial fibrillation with serum urate adjusting for cardiovascular risk factors in Roselli's study.

Model	Direct effect	OR	95% CI	*p* value
Adjusted for BMI	0.022	1.02	0.99–1.05	0.17
Adjusted for LDL‐c	0.017	1.02	0.98–1.06	0.37
Adjusted for CHD	0.017	1.02	0.96–1.07	0.51
Adjusted for hypertension	0.017	1.02	0.98–1.06	0.37
Adjusted for HbA1C	NA	NA	NA	NA
Adjusted for all	0.007	1.00	0.96–1.06	0.77

Abbreviations: BMI, body mass index; CHD, coronary heart disease; HbA1C, hemoglobin A1c; LDL‐c, low‐density lipid cholesterol.

**Table 4:** Multivariable Mendelian randomization associations of atrial fibrillation with serum urate adjusting for cardiovascular risk factors in Nielsen's study.

Model	Direct effect	OR	95% CI	*p* value
Adjusted for BMI	0.0004	1.00	0.98–1.02	0.97
Adjusted for LDL‐c	−0.0044	1.00	0.97–1.02	0.76
Adjusted for CHD	0.0007	1.00	0.98–1.03	0.95
Adjusted for hypertension	0.0037	1.00	0.97–1.04	0.82
Adjusted for HbA1C	0.0041	1.00	0.98–1.03	0.75
Adjusted for all	−0.0097	1.00	0.97–1.03	0.95

Abbreviations: BMI, body mass index; CHD, coronary heart disease; HbA1C, hemoglobin A1c; LDL‐c, low‐density lipid cholesterol.

Sample size and IV‐specific variation in serum urate concentration were considered to calculate the statistical power of the MR study. Using an alpha level of 5%, the effect with statistical significance may be detected with > 95% statistical power using MR analysis if the causative link between serum urate concentration and AF were as pronounced as observed in the epidemiological studies (OR = 1.1 per 1 mg/dL of SU) (Table 5).

**Table 5:** Power calculations for MR analyses of the effect of serum urate on AF.

OR of AF per 1 mg/dL of SU	Sample size	Type‐I error rate	Proportion of cases	*r* ^2^	Power of MR analysis (%)
Roselli's study					
1.1	495 256	0.05	0.11	0.036	96.7
1.2	495 256	0.05	0.11	0.036	100
1.3	495 256	0.05	0.11	0.036	100
Nielsen's study					
1.1	1 000 000	0.05	0.06	0.036	98.7
1.2	1 000 000	0.05	0.06	0.036	100
1.3	1 000 000	0.05	0.06	0.036	100

Abbreviations: AF, atrial fibrillation; OR, odds ratio; SU, serum urate.

## 4 Discussion

In this study, we employed MR methods to assess the causal relationship between genetically predicted serum urate levels and AF. Our findings indicate no causal association between genetically determined serum urate levels and AF, a pattern that remained consistent across two different AF GWAS data sets and did not change after adjusting for potential confounders in multivariable MR analyses.

The innovation of this study lies in its bidirectional MR design, which not only evaluates the potential causal effect of high serum urate levels on AF but also examines the possible reverse causality from AF to serum urate levels. This bidirectional analysis enhances our understanding of the relationship between the two and provides a new perspective for future research. Furthermore, our study capitalizes on a large sample size and multiple data sources, increasing statistical power and allowing for a more precise assessment of potential confounding factors. Through these methods, we provide more robust evidence that there is a lack of significant causal association between serum urate levels and AF, even after adjusting for a variety of cardiovascular risk factors. These findings have important implications for guiding future clinical practice and research, suggesting that therapeutic strategies aimed at lowering serum urate may not reduce the risk of AF.

A prior MR study investigation examined the potential link between serum urate level and the risk of AF but did not demonstrate a significant correlation. Of note, the study sample size (633 participants) was insufficient to yield adequate statistical power for accessing a causal relationship with AF. Moreover, the number of IVs (nine SNPs) was relatively small enough to yield valid results. Besides, the study did not account for pleiotropy [30]. In comparison to prior investigations, this present study benefits from a significantly larger sample size (*N* > 500 000), resulting in a greater level of statistical power. Therefore, the inability to identify the predicted epidemiological connection between serum urate levels and AF through MR is not due to a lack of power. Besides, the association did not change after performing multivariable MR analysis to adjust confounders, yielding more reliable results.

Our findings could be inconsistent with some observational studies that have indicated that increased serum urate levels are independent AF predictors [31], including extremely large population study [32]. However, population‐based association studies cannot prove causation because of confounding factors and reverse causality. In observational studies, the unknown bias and confounders may mask the real association between serum urate level and AF. Moreover, in some studies, the serum urate level was recorded at baseline, significantly changing during follow‐up. Besides, Hashimoto et al. reported that urate‐lowering therapy could not reduce AF risk [33]. MR studies use genetic variants as IV, which are randomly assigned at fertilization and not influenced by traditional confounders such as environmental exposures, socioeconomic status, and behavioral factors. Therefore, our study results may be more capable of excluding the interference of confounding factors and reverse causality. However, MR study also has its limitations. MR evaluates the cumulative, lifelong effect of genetic variations associated with serum UA levels. Therefore, the findings should not be considered as the impact of serum UA at a specific period. Besides, the results of MR studies may not be applicable to all populations or environments, especially if genetic variants have different impacts on different populations. Another reason of the difference between observational study results and MR results may be due to AF type. Different types of AF may have distinct pathophysiological mechanisms and risk factors. Ding's study reported that elevated serum urate increases the risk of new‐onset AF [32], our study did not stratify AF types. When integrating these two sets of results, we suggest combining results from different study designs to strengthen the strength of causal inference. Our MR study results provide a more reliable form of evidence, while observational studies results provide evidence of associations in natural settings. The combination of both may help to more comprehensively understand the relationship between UA and AF.

The key strength of this study lies in its utilization of the MR study design, which enhances the reliability of the causal impact by estimating unbiased causal effects. Additionally, the inclusion of sensitivity studies, which assessed the consistency of primary results using various statistical models and leave‐one‐out analyses, added robustness to the evidence presented. In the meantime, the genetic correlations related to AF were retrieved from different data sources.

Nevertheless, it is necessary to acknowledge certain constraints. To begin, the possibility of horizontal pleiotropy cannot be entirely excluded, which may potentially influence the study outcomes. However, the MR‐PRESSO evaluation did not reveal any indications of such pleiotropy in the results. Second, AF included paroxysmal AF, persistent AF, and permanent AF. Different types of AF did not stratify current GWAS studies. This may confound the correlation between serum UA and other AF types. Stratified analyses depending on AF type and duration are required with the availability of GWAS individual‐level data or more elaborate future GWAS summary data. Different types of AF may have distinct pathophysiological mechanisms and risk factors, and the duration of AF episodes could also influence the association with UA levels. Future studies with access to individual‐level data from GWAS or more detailed GWAS summary statistics are needed to perform these stratified analyses. Such data would allow for a more precise assessment of the relationship between UA and specific subtypes of AF, potentially uncovering associations that are not evident in overall analyses. Additionally, understanding whether UA has a different impact on the initiation versus the progression of AF could have significant clinical implications. It may help tailor preventive and therapeutic strategies more effectively, targeting UA reduction in patient subgroups that might benefit the most. Third, the present research primarily focused on individuals of European ancestry to minimize potential biases related to population structure. Nonetheless, this narrow focus might hamper the applicability of the main findings to other population groups.

## 5 Conclusion

According to the findings of the MR research, there is no evidence of a bidirectional causality link between urate levels and the risk of AF. This implies that treatments aimed at lowering UA may not reduce the risk of AF.

## Author Contributions

Data analysis and manuscript writing: Shandong Yu. Data proofreading and study design: Yanpeng Chu. Revision of the manuscript: Yanpeng Chu, Wei Wang, and Yulian Gao. Data curation and software: Yanpeng Chu and Jun Zhou. All authors reviewed the manuscript.

## Conflicts of Interest

The authors declare no conflicts of interest.

## Supporting information

Supporting information.

## Data Availability

The data that supports the findings of this study are available in the supplementary material of this article. The Weblinks of GWAS data were listed below. Global Urate Genetics Consortium (GUGC) weblink: http://gwas.mrcieu.ac.uk/,ID:ieu-a-1055. Roselli's study weblink: http://www.ebi.ac.uk/gwas/,ID:GCST006061. Nielsen's study weblink: http://www.ebi.ac.uk/gwas/,ID:GCST006414. The Genetic Investigation of Anthropometric Traits (GIANT) weblink: http://gwas.mrcieu.ac.uk/,ID:ieu-a-835. The Meta‐Analyses of Glucose and Insulin‐Related Traits Consortium (MAGIC) weblink: http://gwas.mrcieu.ac.uk/,ID:ieu-b-103. The Global Lipids Genetics Consortium (GLGC) weblink: http://www.ebi.ac.uk/gwas/,ID:GCST002222. Coronary heart disease from CARDIoGRAMplusC4D weblink: http://www.ebi.ac.uk/gwas/,ID:GCST003116.
