# Supplementary material for: Serum Urate and Atrial Fibrillation: A Bidirectional Mendelian Randomization Study
Source: Clin Cardiol. 2025 Jan 27;48(1):e70089. doi: 10.1002/clc.70089 (PMC11772717; doi:10.1002/clc.70089)
Supplement: Supplementary file 1 — Supporting information. [file CLC-48-e70089-s001.pdf]

Supplemental Figure 1

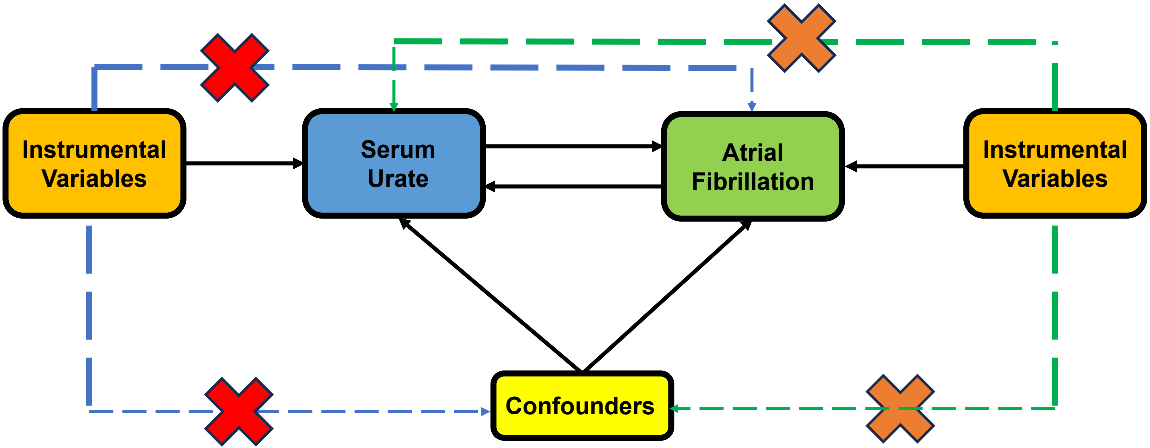

Supplemental Figure 2

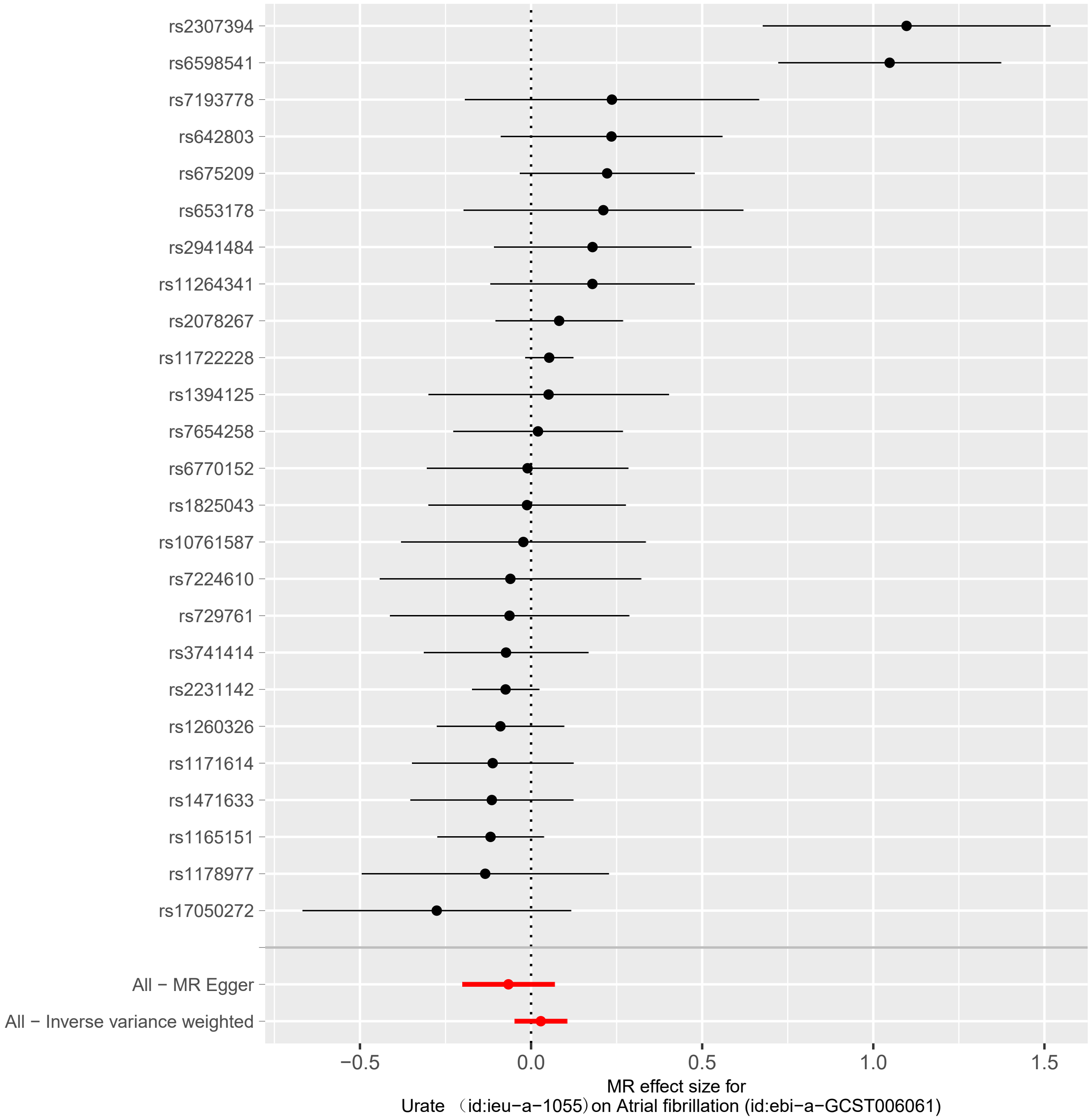

Supplemental Figure 3

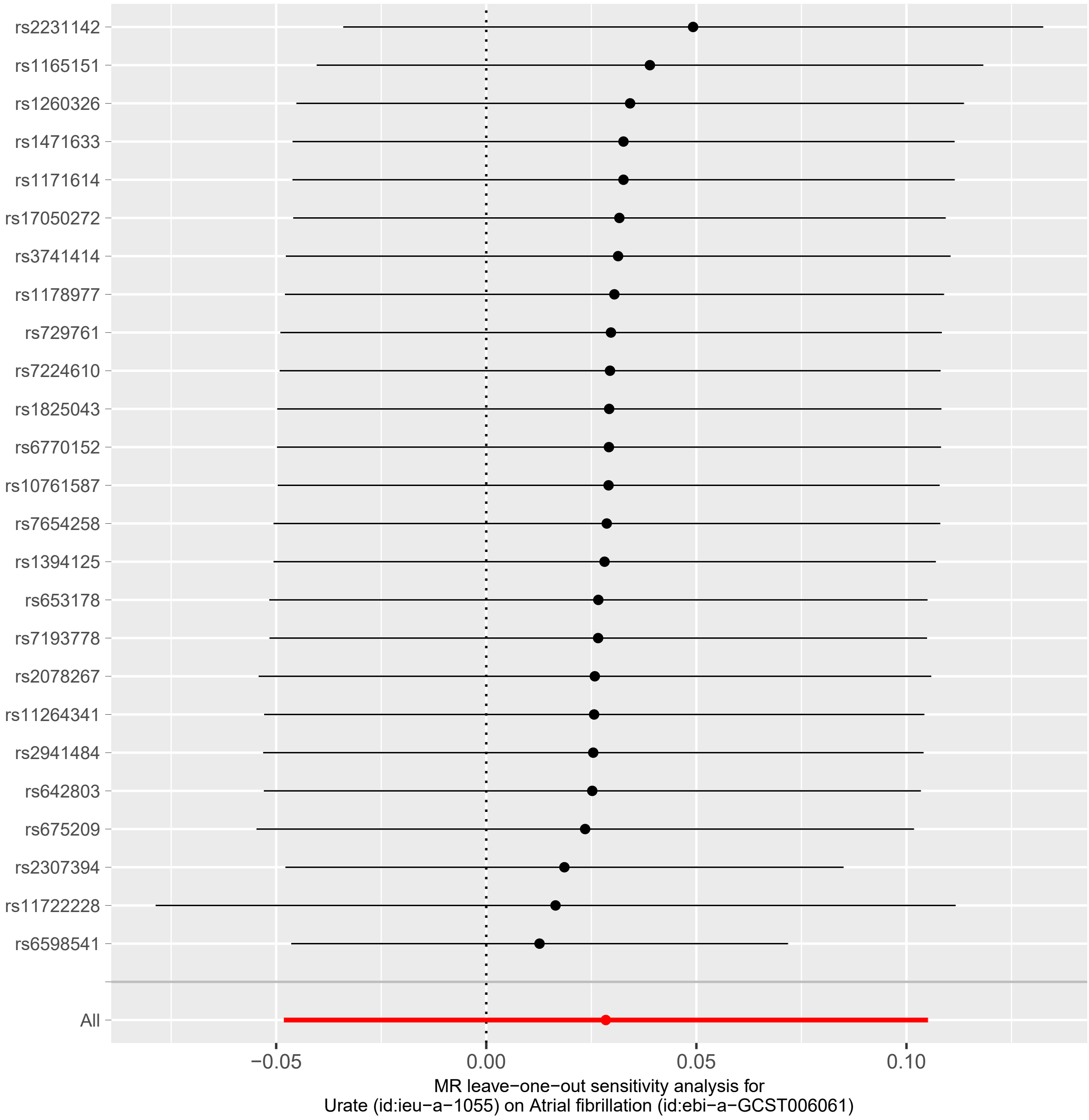

Supplemental Figure 4

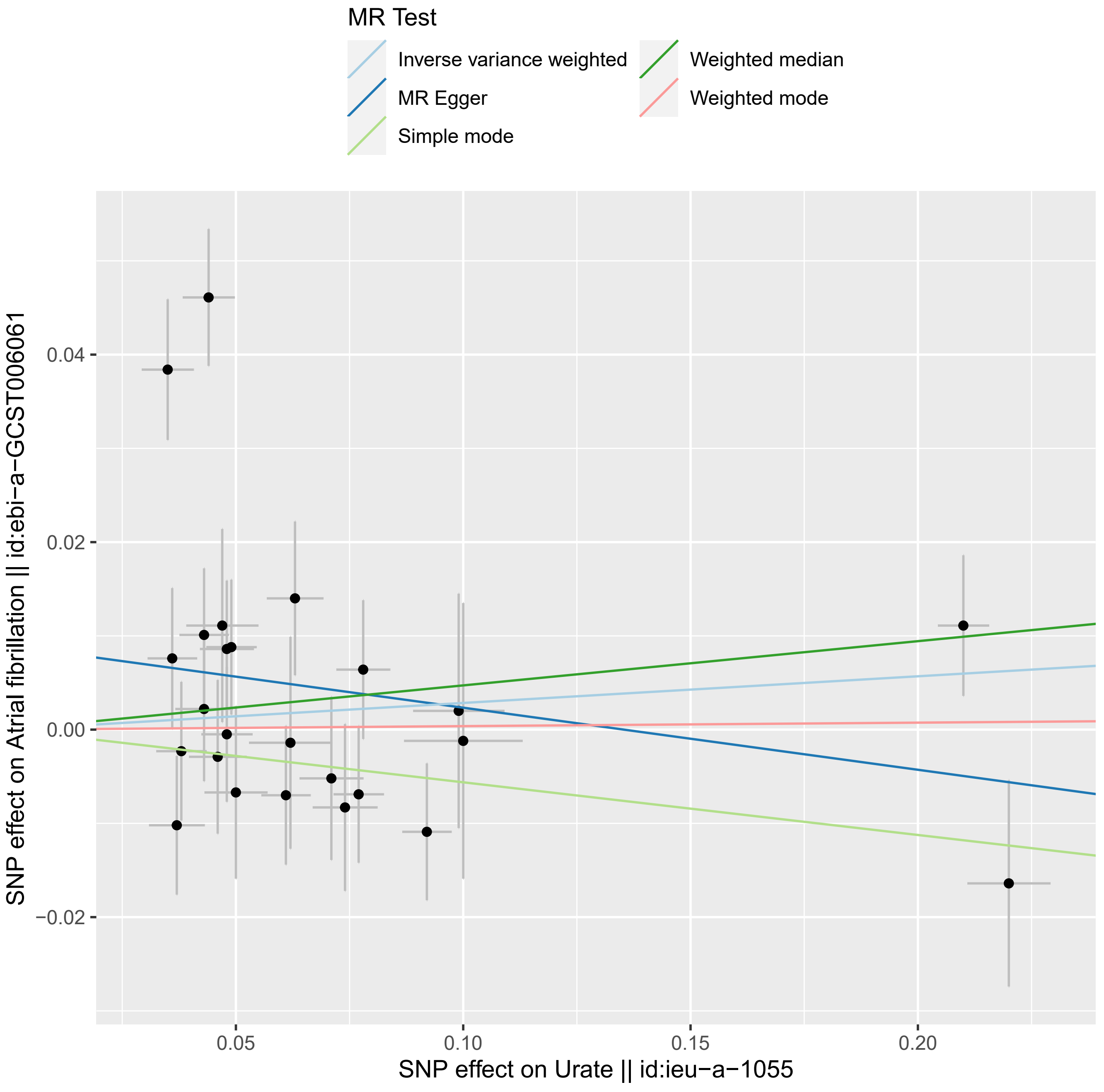

Supplemental Figure 5

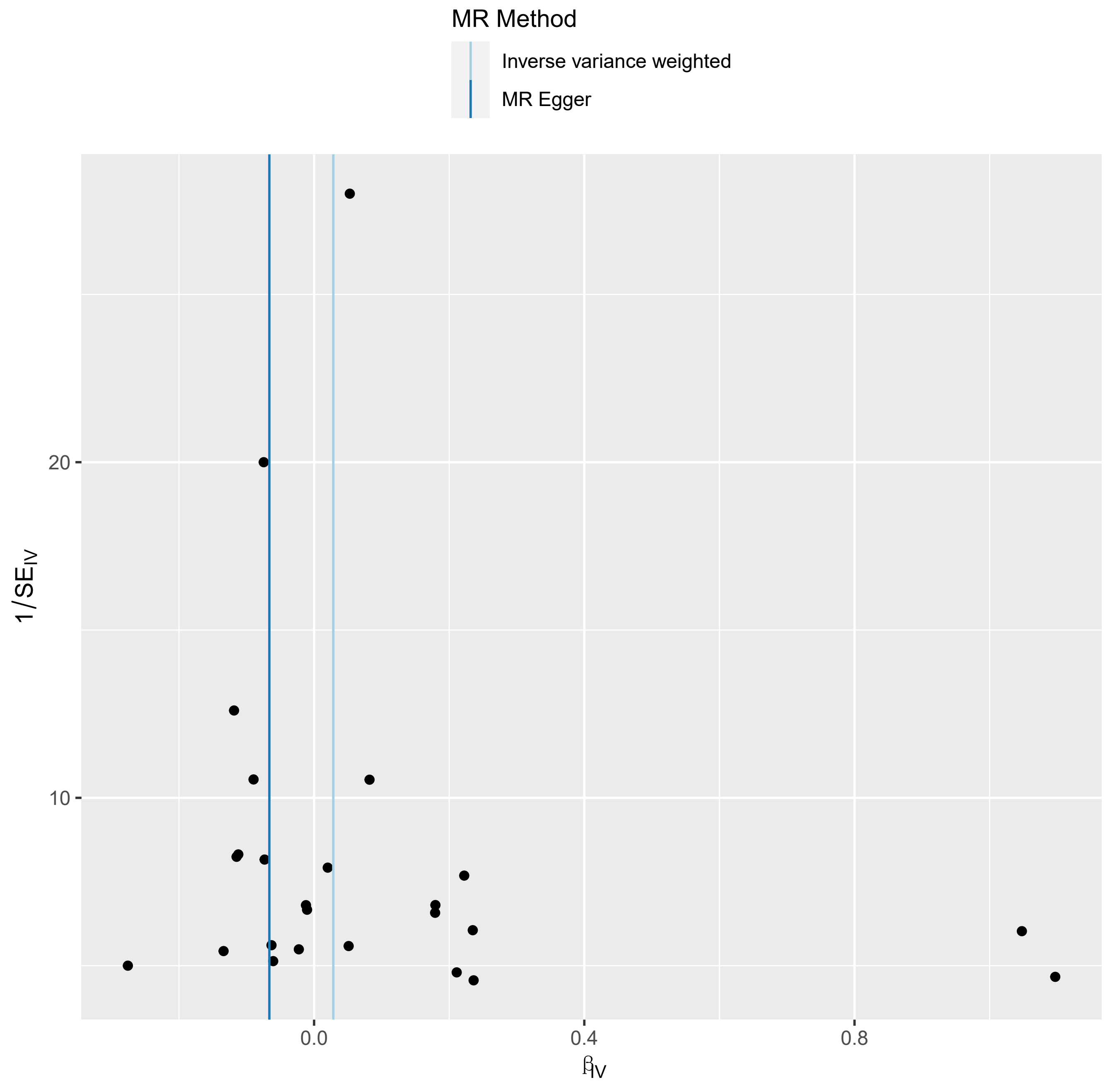

Supplemental Figure 6

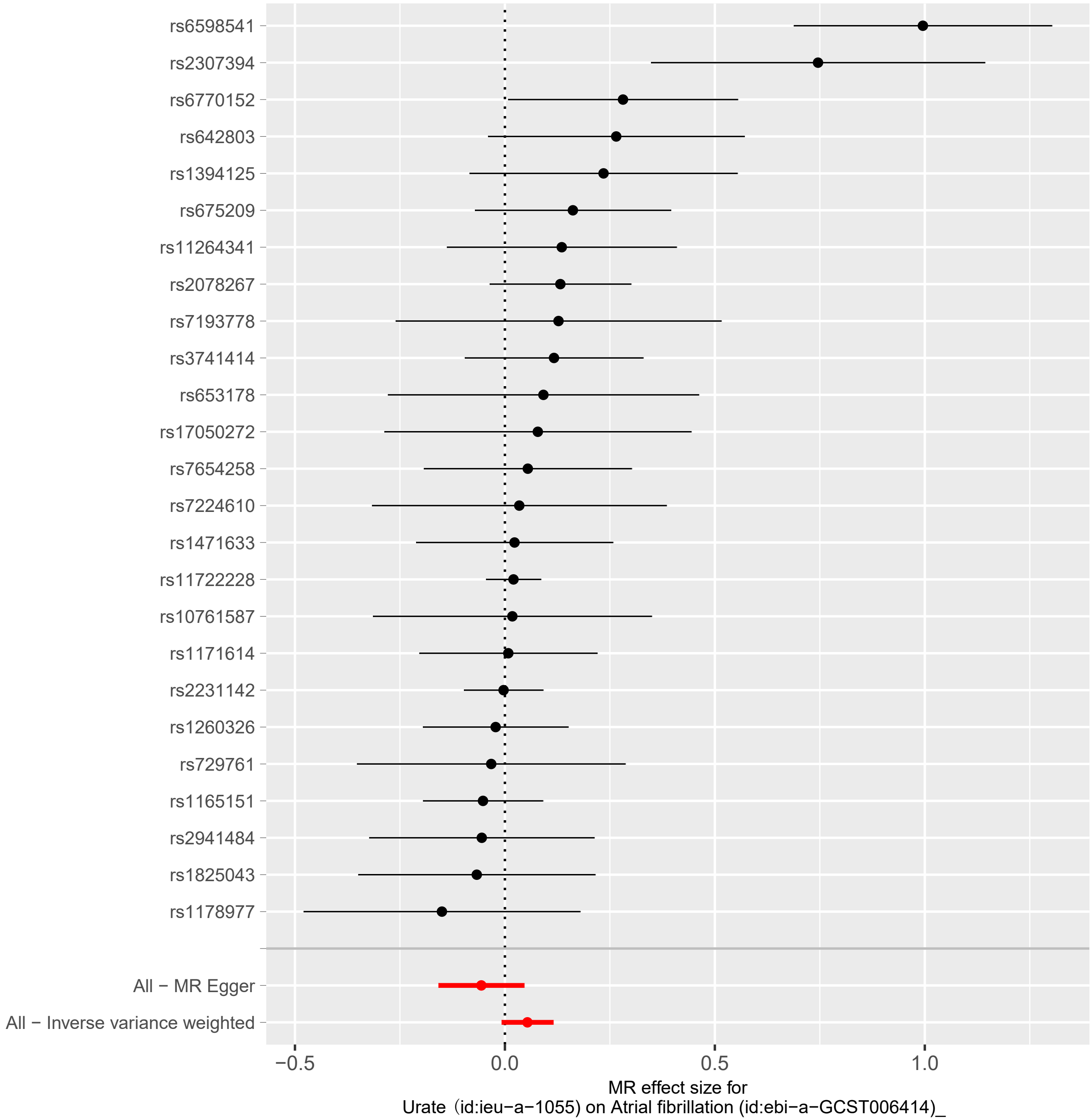

Supplemental Figure 7

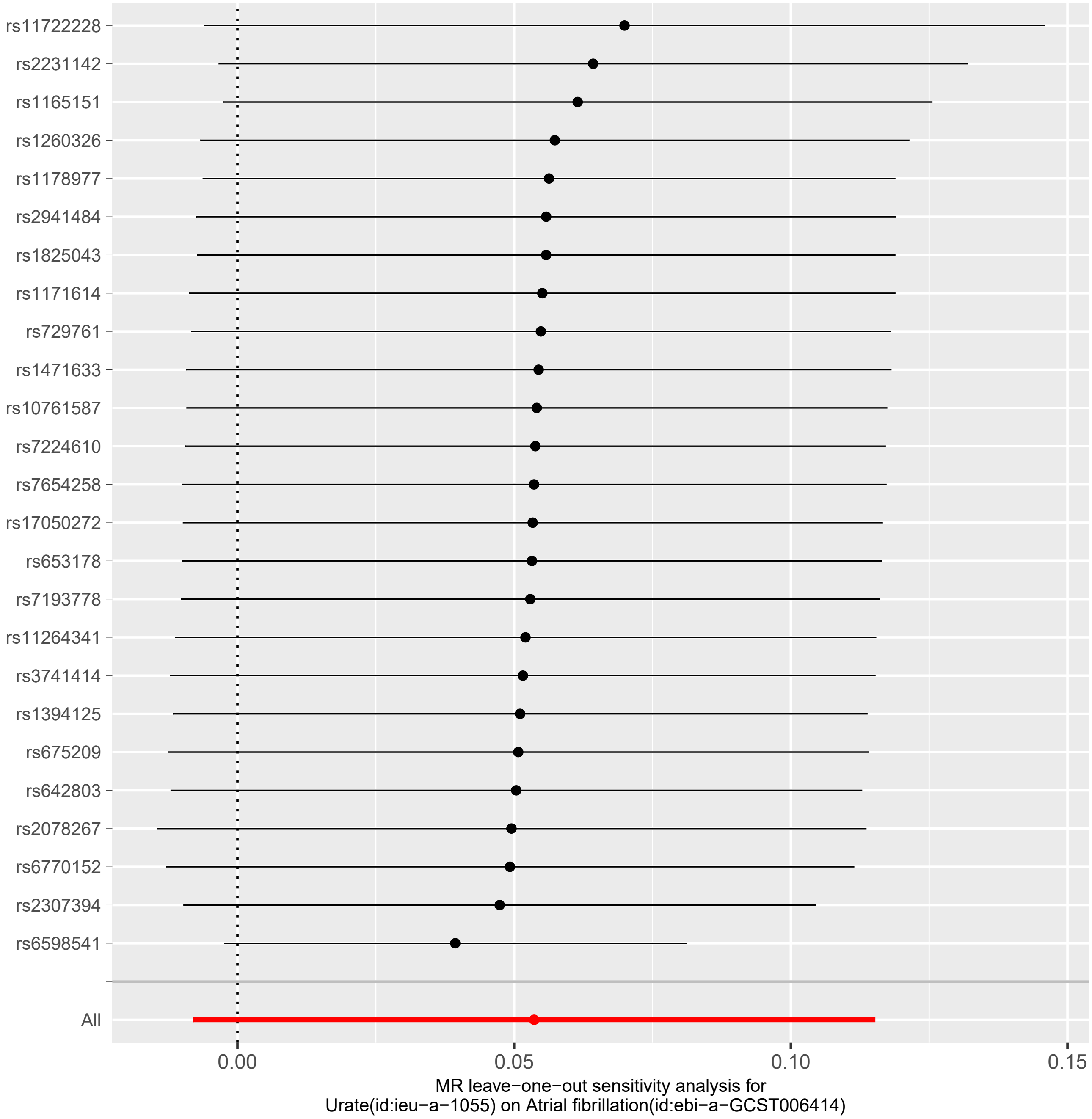

Supplemental Figure 8

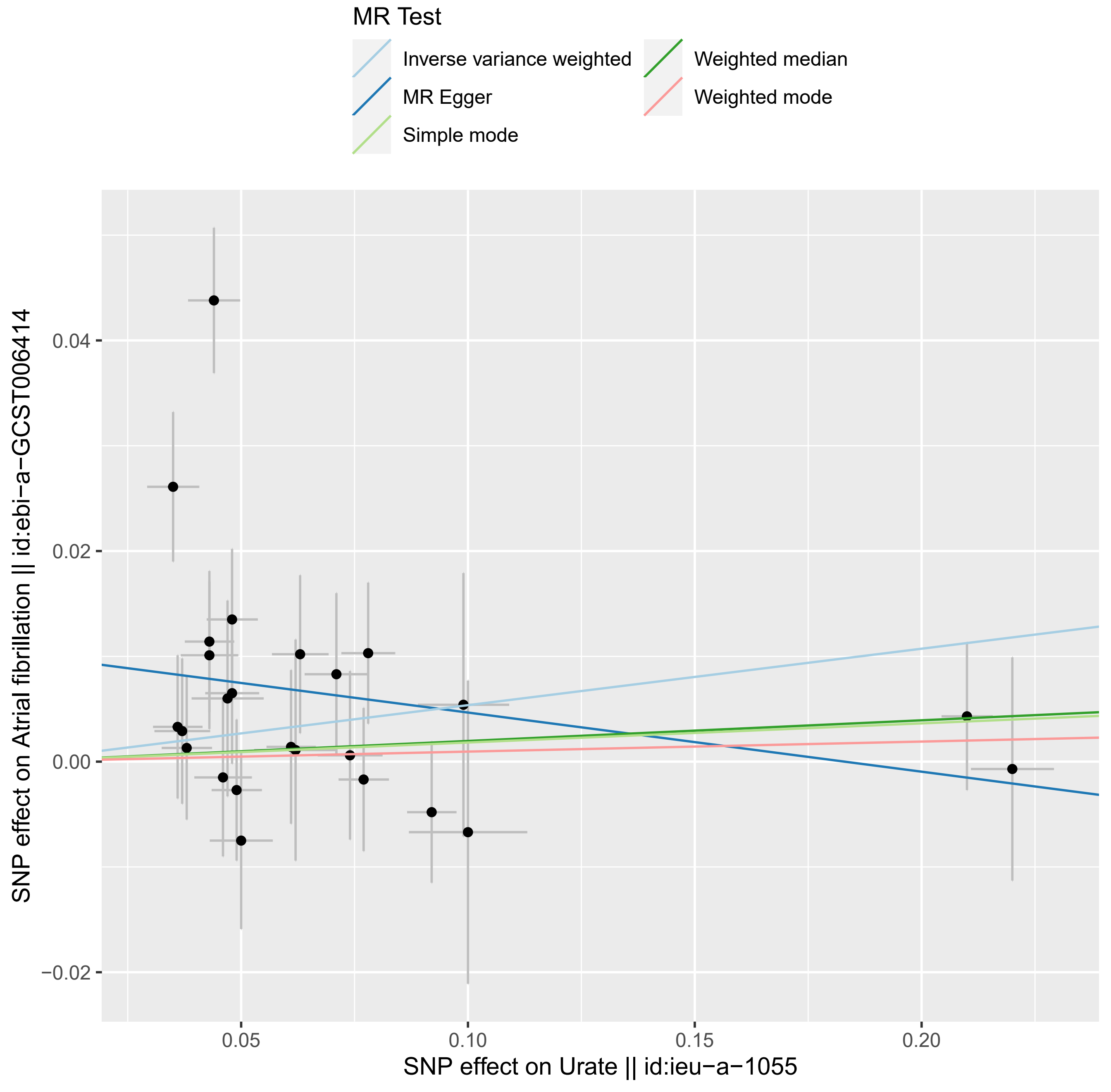

Supplemental Figure 9

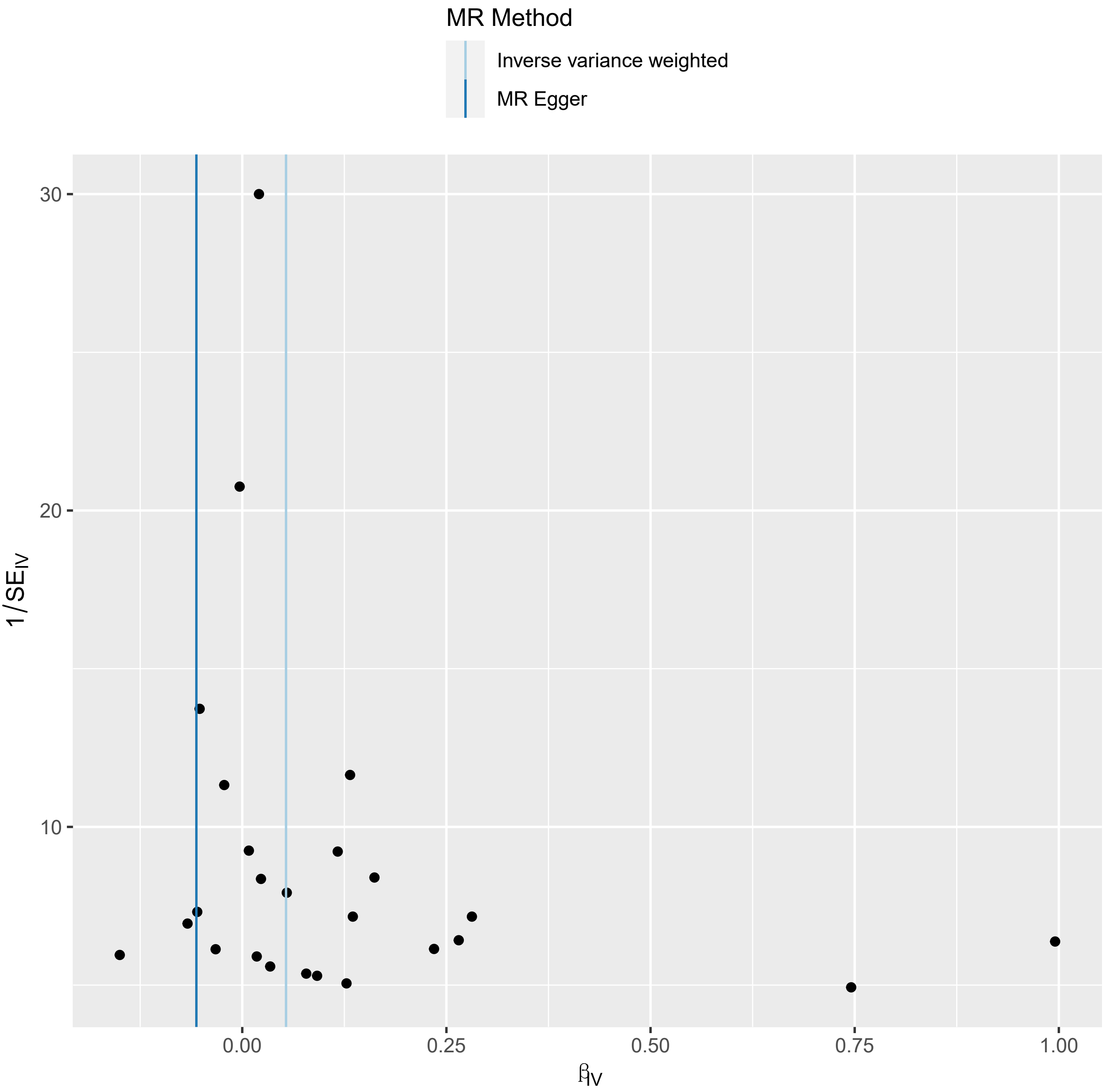

Supplemental Figure 10

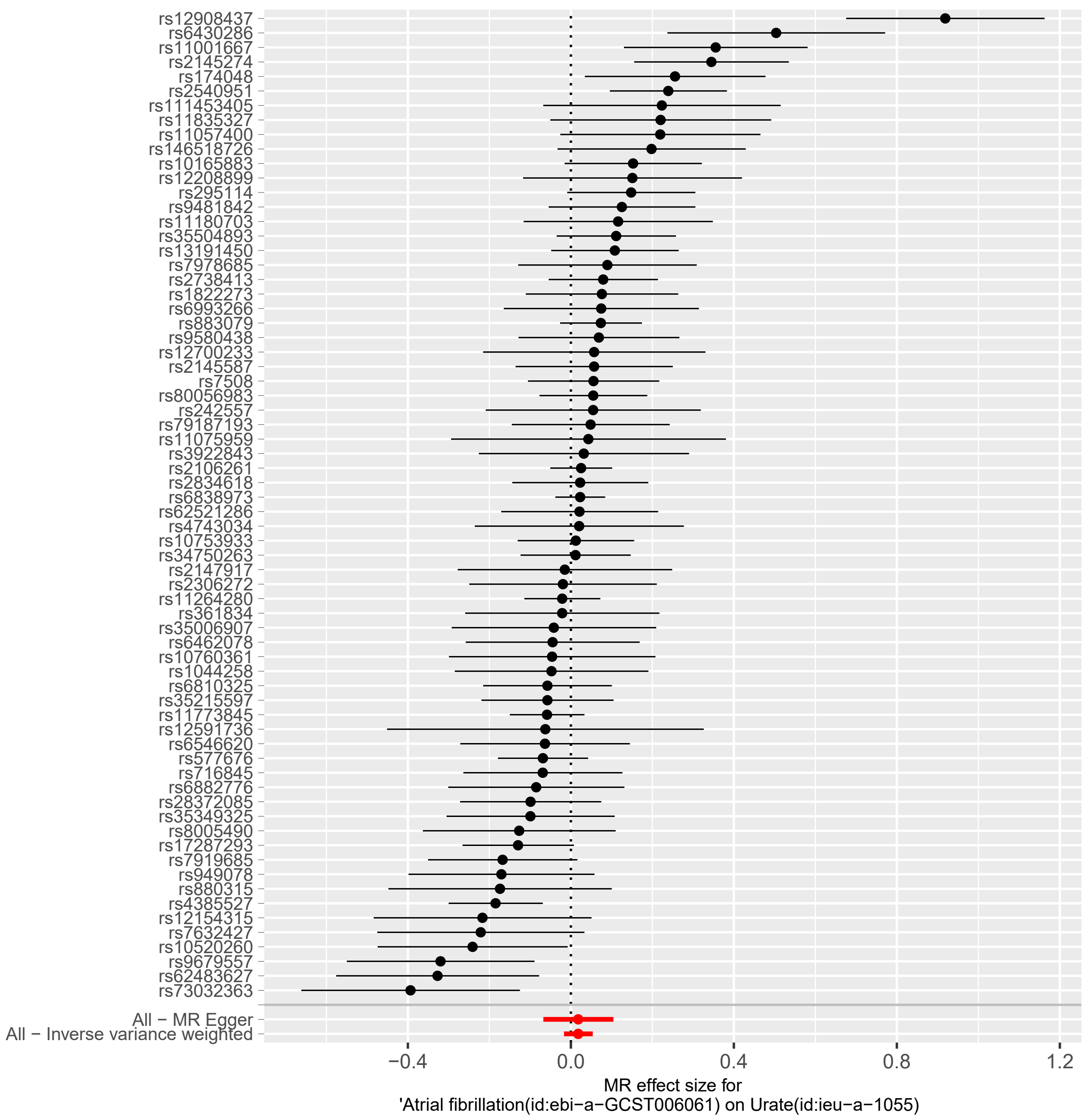

Supplemental Figure 11

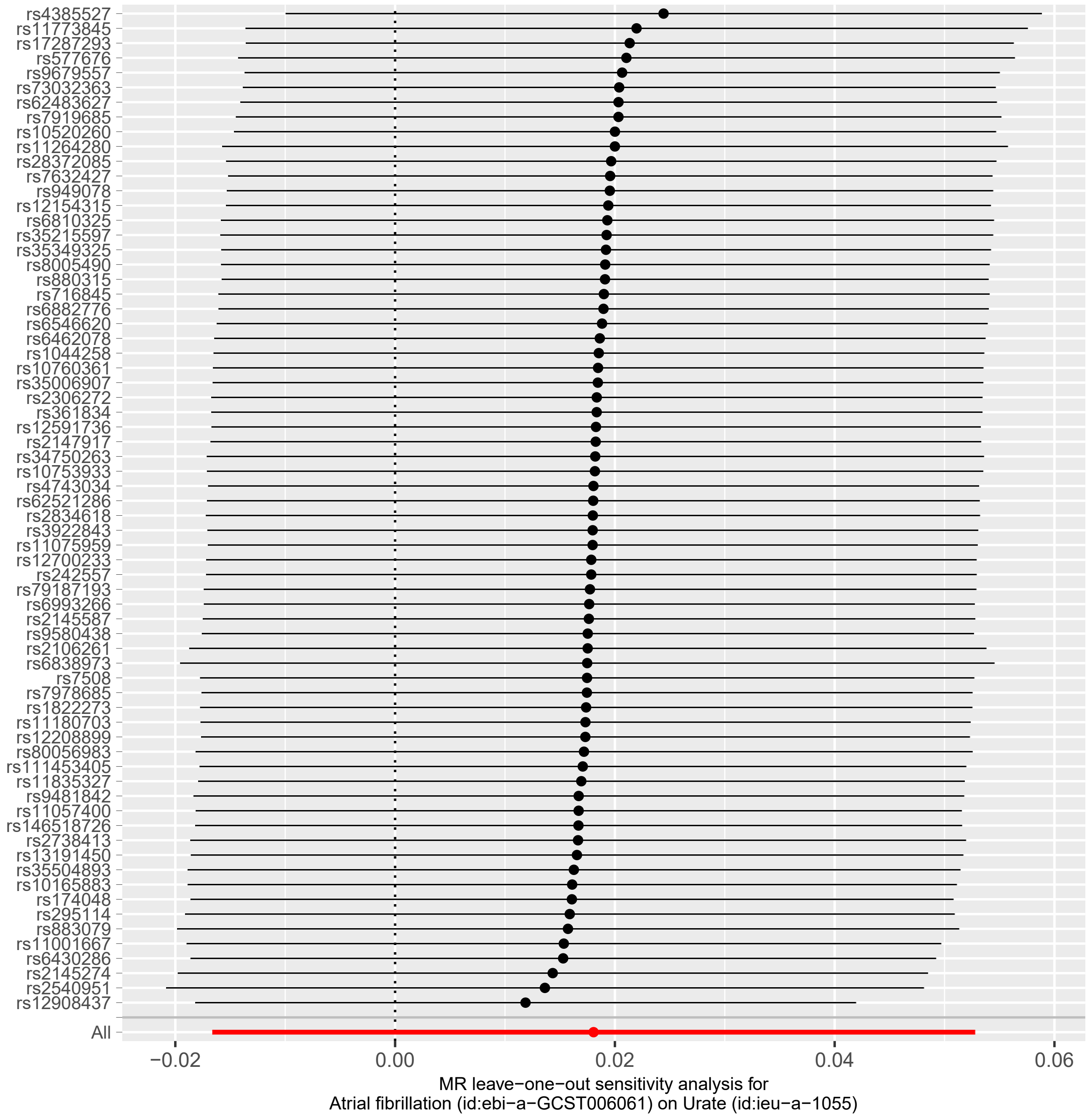

Supplemental Figure 12

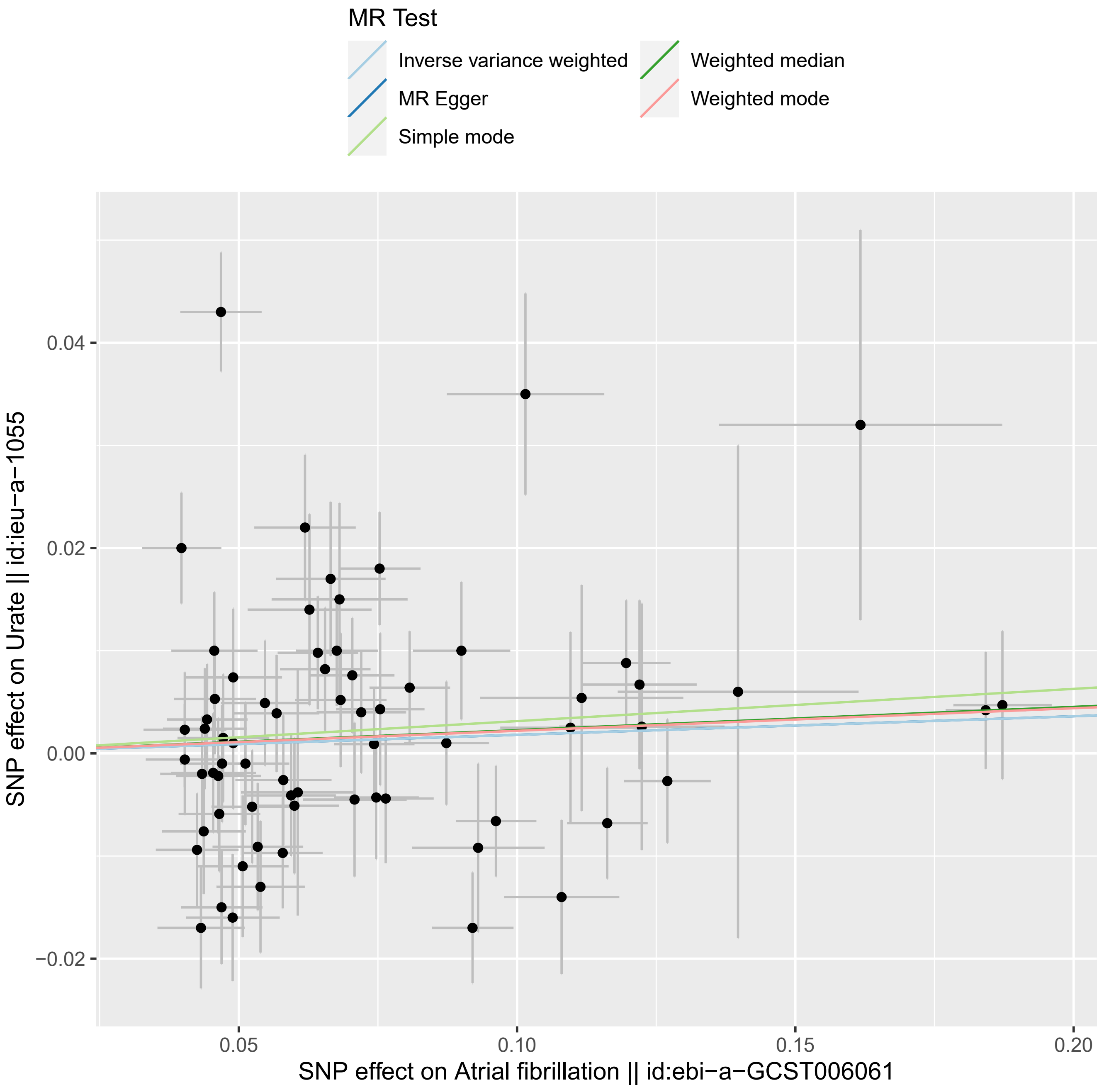

Supplemental Figure 13

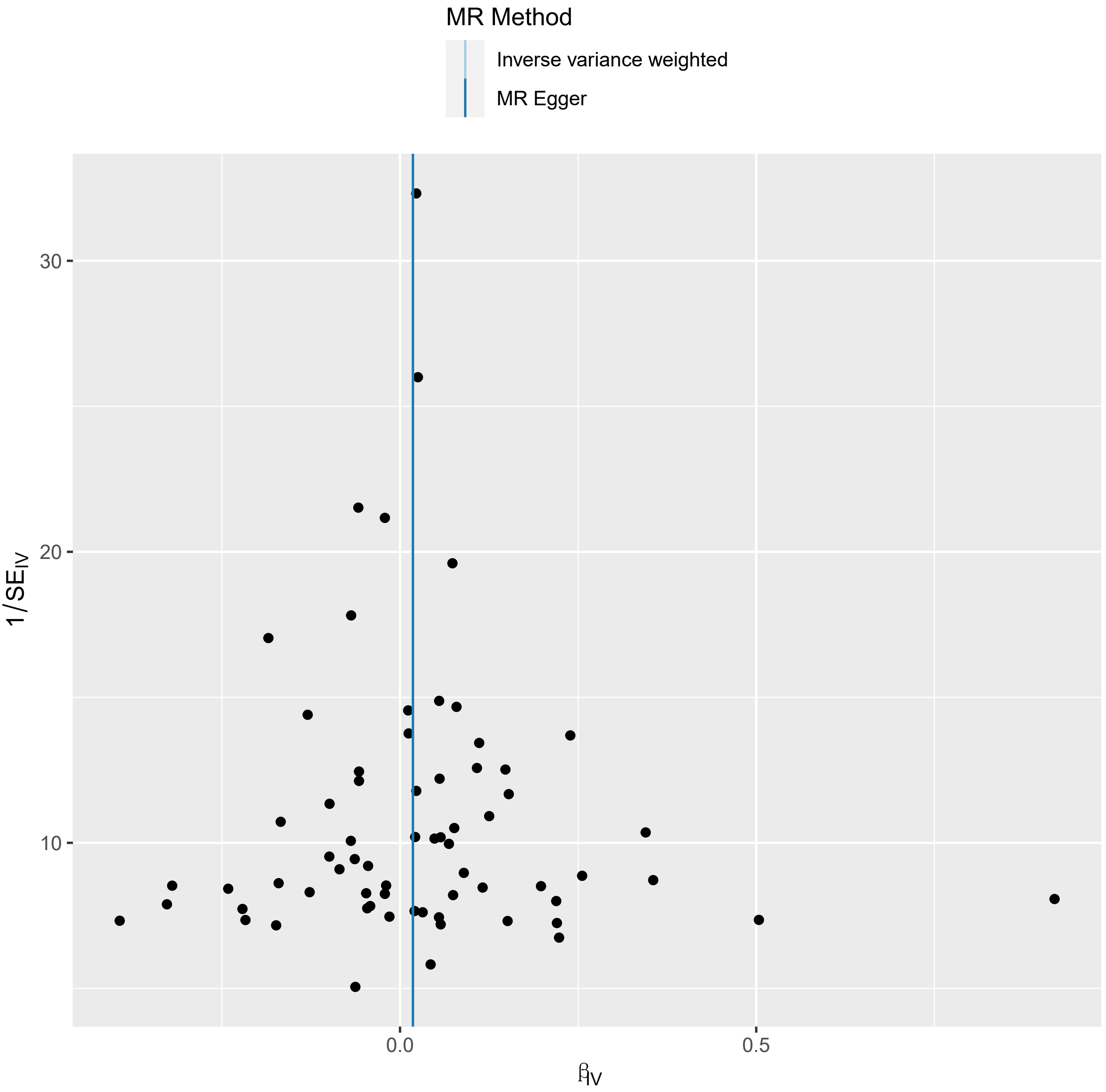

Supplemental Figure 14

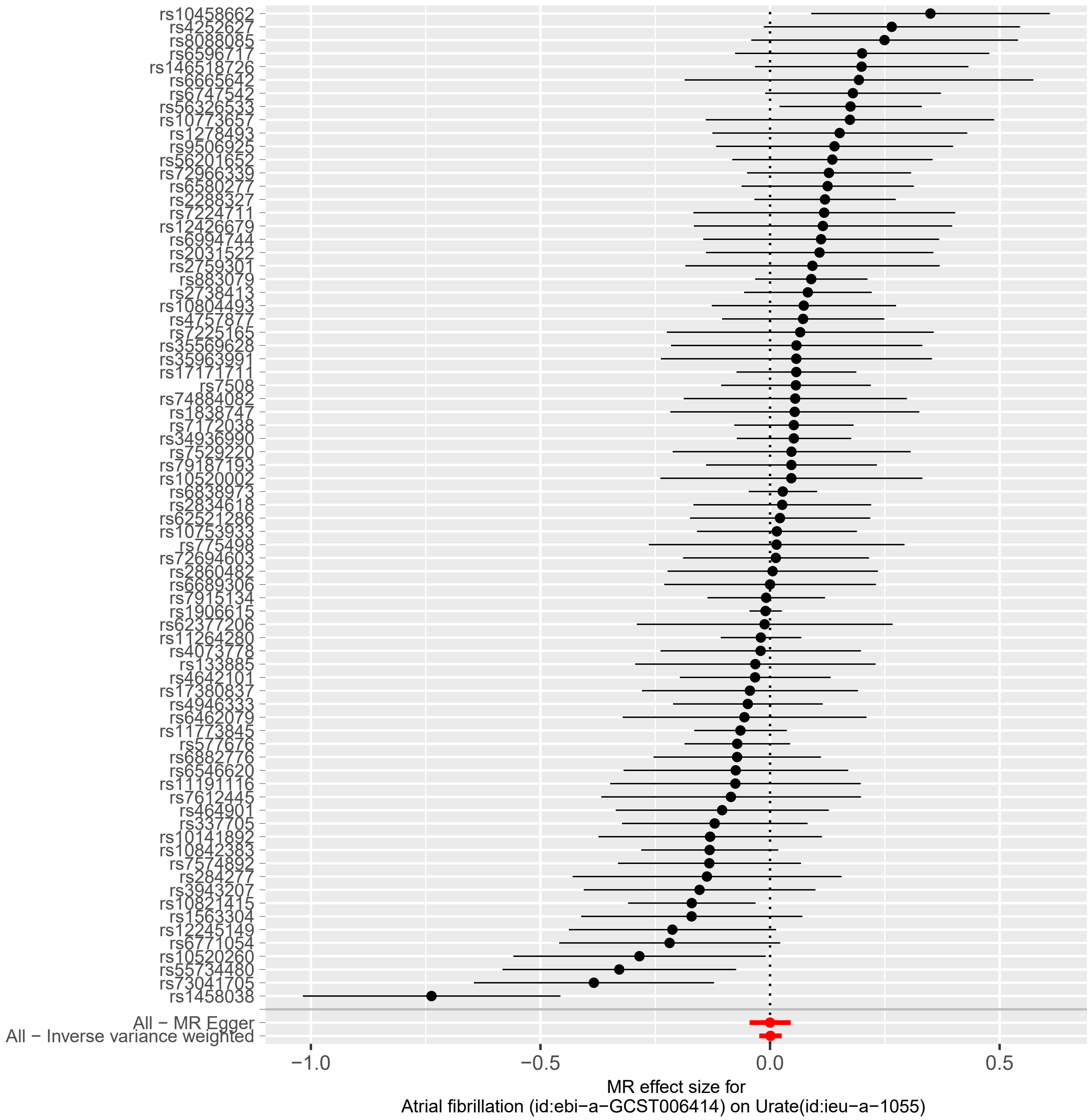

Supplemental Figure 15

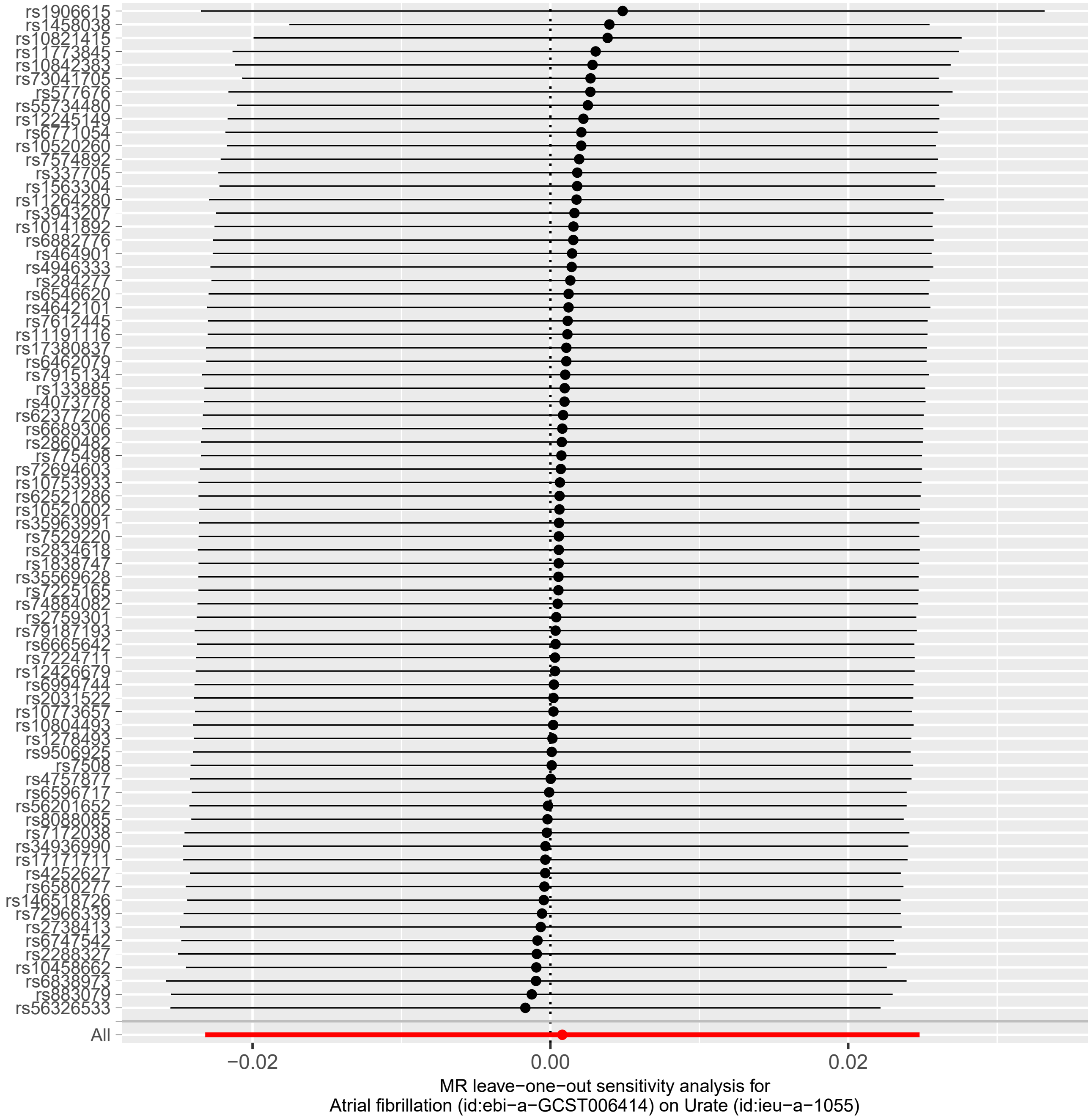

Supplemental Figure 16

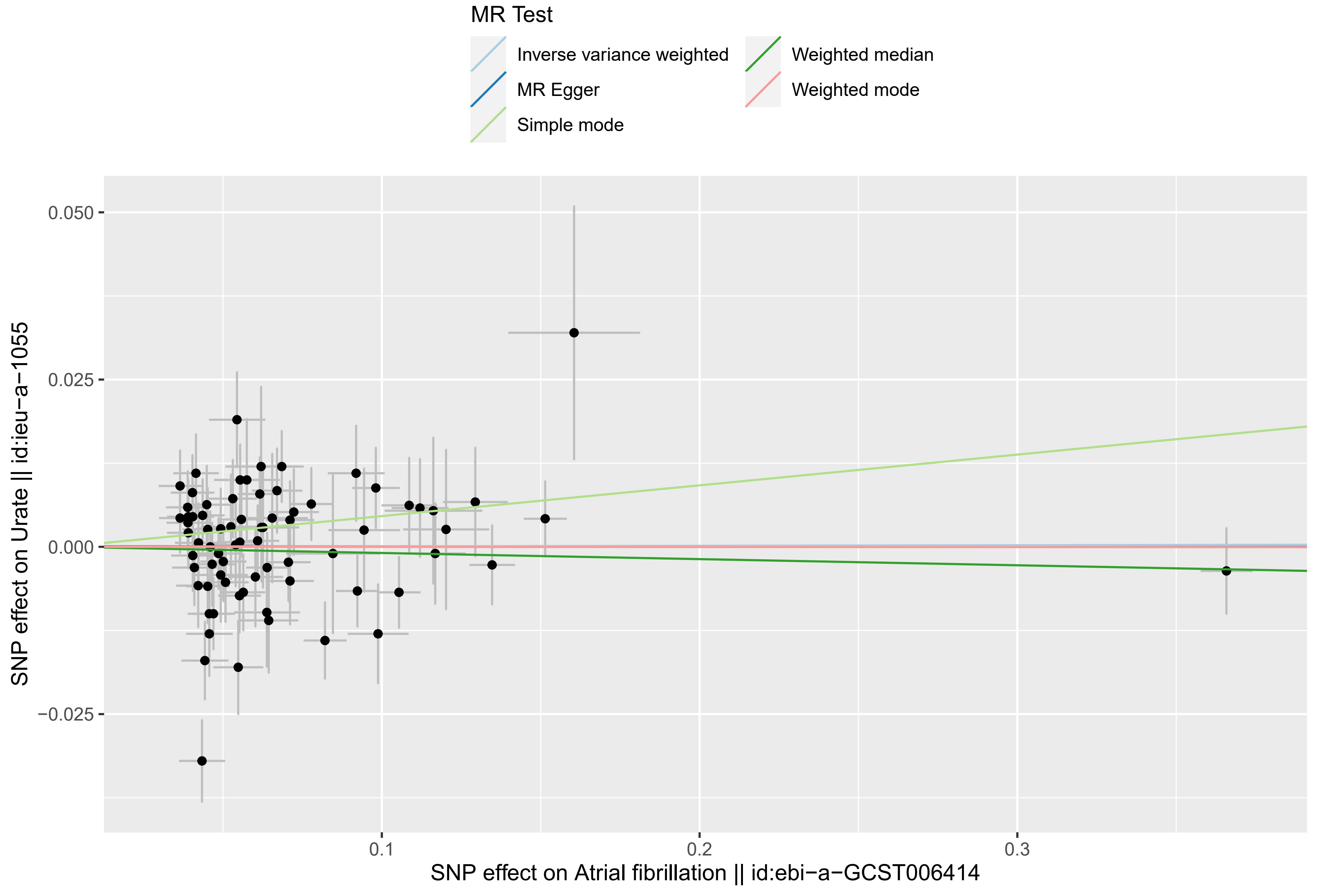

Supplemental Figure 17

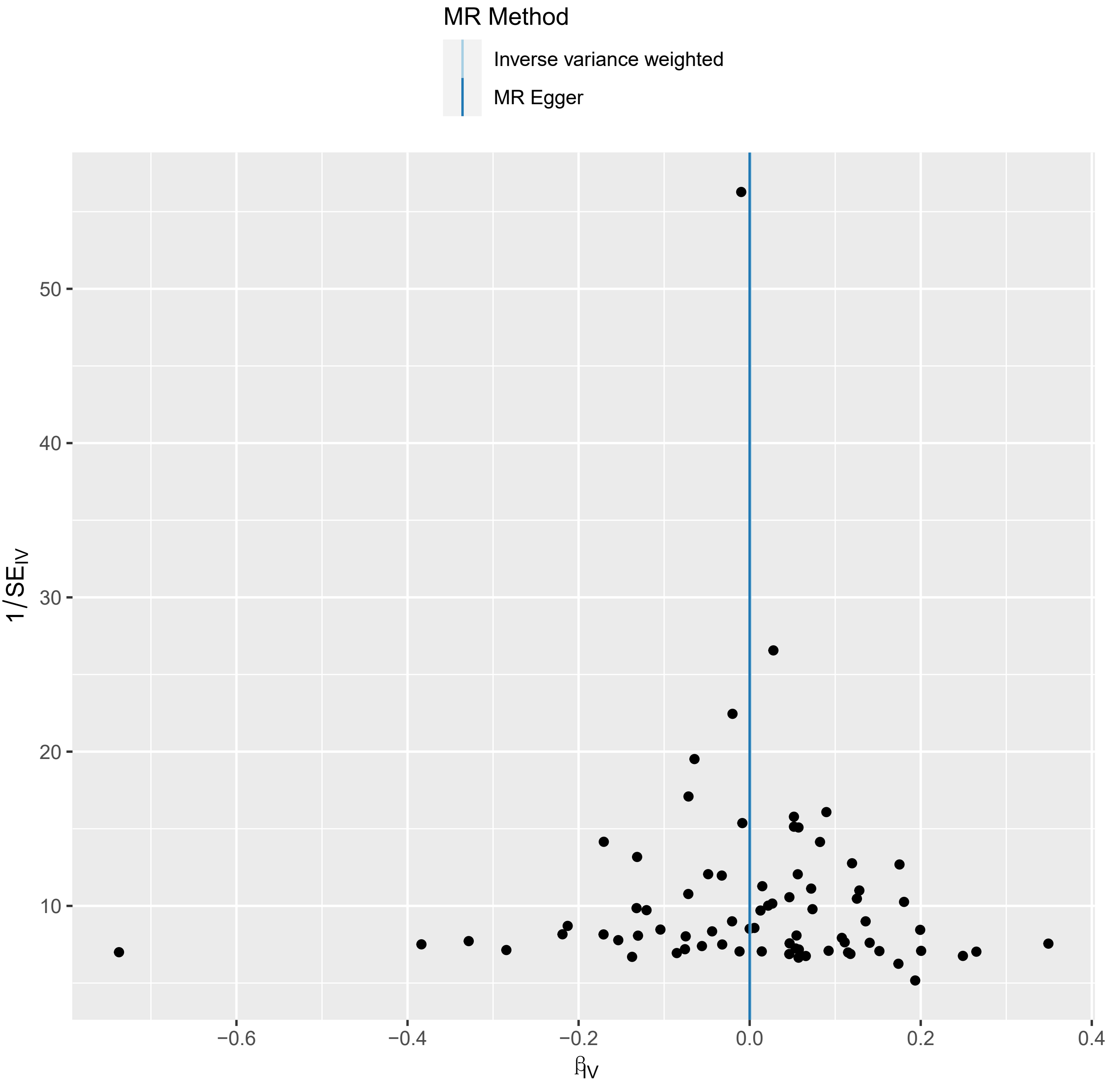

Supplemental Table 1. Genome-wide significant (p<5x10-8) single nucleotide polymorphisms that were used as instruments for serum urate level.

Exposure	SNP	Chr	EA	NEA	EAF	P-value	Beta	Se	N	R^2	F value
Urate	rs11264341	1	T	C	0.43	1.00E-14	-0.048	0.006	105030	6.09E-04	63.9987813
Urate	rs1471633	1	A	C	0.46	1.40E-26	0.061	0.005	108616	1.37E-03	148.8372593
Urate	rs1260326	2	T	C	0.41	1.30E-40	0.077	0.006	110195	1.49E-03	164.6914553
Urate	rs17050272	2	A	G	0.43	9.40E-09	0.037	0.006	103514	3.67E-04	38.02704304
Urate	rs2307394	2	T	C	0.68	7.30E-09	-0.035	0.006	109642	3.10E-04	34.02715707
Urate	rs6770152	3	T	G	0.58	2.70E-16	-0.048	0.006	109889	5.82E-04	63.99883519
Urate	rs11722228	4	T	C	0.36	3.30E-261	0.206	0.006	110052	1.06E-02	1178.756356
Urate	rs1825043	4	A	G	0.95	6.70E-14	0.1	0.013	109896	5.38E-04	59.17052077
Urate	rs2231142	4	T	G	0.11	4.40E-116	0.221	0.009	110093	5.45E-03	602.9643547
Urate	rs6830367	4	C	G	0.18	2.40E-11	0.051	0.007	109881	4.83E-04	53.08066649
Urate	rs7654258	4	T	C	0.91	1.10E-19	-0.099	0.01	105384	9.29E-04	98.00813995

Urate	rs17632159	5	C	G	0.31	2.00E-09	-0.038	0.006	108153	3.71E-04	40.11036936
Urate	rs1165151	6	T	G	0.47	4.50E-60	-0.093	0.005	109945	3.14E-03	345.9537067
Urate	rs675209	6	T	C	0.27	1.40E-21	0.063	0.006	109893	1.00E-03	110.2479935
Urate	rs729761	6	T	G	0.30	3.1E-12	-0.046	0.006	108706	4.99E-04	54.24339605
Urate	rs1178977	7	A	G	0.81	6.70E-12	0.05	0.007	109469	4.66E-04	51.01947602
Urate	rs2941484	8	T	C	0.44	3.90E-17	0.049	0.006	109903	6.06E-04	66.69323075
Urate	rs10761587	10	T	C	0.11	9.60E-11	0.062	0.009	107891	4.40E-04	47.45591041
Urate	rs1171614	10	T	C	0.22	6.50E-23	-0.074	0.007	103697	1.08E-03	111.7529466
Urate	rs2078267	11	T	C	0.51	8.70E-36	-0.078	0.006	97905	1.72E-03	168.9965477
Urate	rs642803	11	T	C	0.46	4.50E-14	-0.043	0.005	109895	6.73E-04	73.95865399
Urate	rs3741414	12	T	C	0.24	9.80E-22	-0.071	0.007	102628	1.00E-03	102.8755462
Urate	rs653178	12	T	C	0.51	2.40E-10	-0.036	0.005	110119	4.71E-04	51.83905847
Urate	rs1394125	15	A	G	0.34	9.80E-11	0.043	0.006	105463	4.87E-04	51.3601371
Urate	rs6598541	15	A	G	0.36	5.20E-13	0.044	0.006	109224	4.92E-04	53.77679305

Urate	rs7193778	16	T	C	0.86	2.40E-08	-0.047	0.008	109915	3.14E-04	34.51499696
Urate	rs7224610	17	A	C	0.58	4.70E-11	-0.038	0.006	110044	3.64E-04	40.11038211

Note: SNP, single nucleotide polymorphism; Chr, chromosome; EA, effect allele; NEA, none effect allele; EAF, effect allele frequence; Se, standard error.

Supplemental Table 2. Genetic associations for serum urate and AF in Roselli's study.

SNP	beta.exposure	se.exposure	beta.outcome	se.outcome
rs10761587	-0.062	0.0091	0.0014	0.0113
rs11264341	-0.048	0.0059	-0.0086	0.0073
rs1165151	0.092	0.0054	-0.0109	0.0073
rs1171614	0.074	0.0071	-0.0083	0.0089
rs11722228	0.21	0.0056	0.0111	0.0075
rs1178977	-0.05	0.0069	0.0067	0.0092
rs1260326	-0.077	0.0055	0.0069	0.0073
rs1394125	0.043	0.0063	0.0022	0.0077
rs1471633	-0.061	0.0054	0.007	0.0074
rs17050272	0.037	0.0061	-0.0102	0.0074
rs17632159	-0.038	0.0061	0.0272	0.0078
rs1825043	0.1	0.013	-0.0012	0.0147
rs2078267	-0.078	0.0059	-0.0064	0.0074
rs2231142	0.22	0.0091	-0.0164	0.011
rs2307394	0.035	0.0057	0.0384	0.0075
rs2941484	0.049	0.0055	0.0088	0.0072
rs3741414	-0.071	0.007	0.0052	0.0087
rs642803	-0.043	0.0054	-0.0101	0.0071
rs653178	-0.036	0.0054	-0.0076	0.0075
rs6598541	-0.044	0.0057	-0.0461	0.0073
rs675209	-0.063	0.0062	-0.014	0.0082
rs6770152	-0.048	0.0056	5.00E-04	0.0072
rs6830367	0.051	0.0072	0.0047	0.0098
rs7193778	-0.047	0.0079	-0.0111	0.0103
rs7224610	-0.038	0.0055	0.0023	0.0074
rs729761	0.046	0.0063	-0.0029	0.0082
rs7654258	-0.099	0.01	-0.002	0.0125

AF, atrial fibrillation; SNP, single nucleotide polymorphism; Se, standard error.

Supplemental Table 3. Genetic associations for serum urate and AF in Nielsen's study.

SNP	beta.exposure	se.exposure	beta.outcome	se.outcome
rs10761587	-0.062	0.0091	-0.0011	0.0105
rs11264341	-0.048	0.0059	-0.0065	0.0067
rs1165151	0.092	0.0054	-0.0048	0.0067
rs1171614	0.074	0.0071	6.00E-04	0.008
rs11722228	0.21	0.0056	0.0043	0.007
rs1178977	-0.05	0.0069	0.0075	0.0084
rs1260326	-0.077	0.0055	0.0017	0.0068
rs1394125	0.043	0.0063	0.0101	0.007
rs1471633	-0.061	0.0054	-0.0014	0.0073
rs17050272	0.037	0.0061	0.0029	0.0069
rs17632159	-0.038	0.0061	0.0211	0.0073
rs1825043	0.1	0.013	-0.0067	0.0144
rs2078267	-0.078	0.0059	-0.0103	0.0067
rs2231142	0.22	0.0091	-7.00E-04	0.0106
rs2307394	0.035	0.0057	0.0261	0.0071
rs2941484	0.049	0.0055	-0.0027	0.0067
rs3741414	-0.071	0.007	-0.0083	0.0077
rs642803	-0.043	0.0054	-0.0114	0.0067
rs653178	-0.036	0.0054	-0.0033	0.0068
rs6598541	-0.044	0.0057	-0.0438	0.0069
rs675209	-0.063	0.0062	-0.0102	0.0075
rs6770152	-0.048	0.0056	-0.0135	0.0067
rs6830367	0.051	0.0072	-0.0121	0.0088
rs7193778	-0.047	0.0079	-0.006	0.0093
rs7224610	-0.038	0.0055	-0.0013	0.0068
rs729761	0.046	0.0063	-0.0015	0.0075
rs7654258	-0.099	0.01	-0.0054	0.0125

AF, atrial fibrillation; SNP, single nucleotide polymorphism; Se, standard error.

Supplemental Table 4. Mendelian randomization association of genetically predicted serum urate level with AF in Roselli's study

Statistical Method	N(snp)	OR	LCI	UCI	P
Weighted Mode	27	1.00	0.94	1.07	0.70
Weighted Median	27	1.05	0.99	1.12	0.14
Simple Mode	27	0.95	0.85	1.06	0.33
MR Egger	27	0.94	0.82	1.07	0.35
Inverse Variance Weighted	27	1.03	0.95	1.11	0.47
MR PRESSO（raw）	27	1.00	0.96	1.05	0.91
MR PRESSO（3 outlier-corrected）	24	1.03	0.95	1.11	0.47

Supplemental Table 5. Mendelian randomization association of genetically predicted serum urate level with AF in the Nielsen's study

Statistical Method	N(snp)	OR	LCI	UCI	P
MR Egger	27	0.95	0.85	1.05	0.29
Weighted median	27	1.02	0.97	1.08	0.47
Inverse variance weighted	27	1.06	0.99	1.12	0.09
Simple mode	27	1.02	0.93	1.11	0.68
Weighted mode	27	1.01	0.96	1.06	0.70
MR PRESSO	27	1.05	0.99	1.12	0.10
MR PRESSO(2 outlier-corrected)	25	1.03	1.00	1.07	0.05

Supplemental Table 6. Mendelian randomization association of genetically predicted AF with serum urate level in the Roselli's study

Statistical Method	N(snp)	OR	LCI	UCI	P
Weighted Mode	68	1.02	0.98	1.07	0.30
Weighted Median	68	1.02	0.99	1.06	0.19
Simple Mode	68	1.03	0.96	1.11	0.40
MR Egger	68	1.02	0.93	1.11	0.68
Inverse Variance Weighted	68	1.02	0.98	1.05	0.31
MR PRESSO（raw）	68	1.02	0.98	1.05	0.31
MR PRESSO（3 outlier-corrected）	65	1.02	0.99	1.04	0.30

Supplemental Table 7. Mendelian randomization association of genetically predicted AF with serum urate level in the Nielsen's study

Statistical Method	nsnp	OR	LCI	UCI	P
MR Egger	75	1	0.96	1.05	1.00
Weighted median	75	0.99	0.96	1.02	0.56
Inverse variance weighted	75	1	0.98	1.03	0.95
Simple mode	75	1.05	0.98	1.12	0.16
Weighted mode	75	1	0.97	1.03	0.99
MR PRESSO	75	1	0.79	1.27	0.95
MR PRESSO(1 outlier-corrected)	74	1	0.81	1.25	0.72

Supplemental Figure Legends

Supplemental Figure 1. Design of the current two-sample bidirectional Mendelian randomization study. Three core assumptions were as follows: (i) relevance assumption; (ii) independence assumption; (iii) exclusion restriction.

Supplemental Figure 2. The forest plot for the association between serum urate and AF in Roselli's study.

Supplemental Figure 3. The leave-one-out analysis for the association between serum urate and AF in Roselli's study.

Supplemental Figure 4. The scatter plot for the association between serum urate and AF in Roselli's study.

Supplemental Figure 5. The funnel plot for the association between serum urate and AF in Roselli's study.

Supplemental Figure 6. The forest plot for the association between serum urate and AF in Nielsen's study.

Supplemental Figure 7. The leave-one-out analysis for the association between serum urate and AF in Nielsen's study.

Supplemental Figure 8. The scatter plot for the association between serum urate and AF in Nielsen's study.

Supplemental Figure 9. The funnel plot for the association between serum urate and AF in Nielsen's study.

Supplemental Figure 10. The forest plot for the association between AF and serum urate in Roselli's study.

Supplemental Figure 11. The leave-one-out analysis for the association between AF and serum urate in Roselli's study.

Supplemental Figure 12. The scatter plot for the association between AF and serum urate in Roselli's study.

Supplemental Figure 13. The funnel plot for the association between AF and serum urate in Roselli's study.

Supplemental Figure 14. The forest plot for the association between AF and serum urate in Nielsen's study.

Supplemental Figure 15. The leave-one-out analysis for the association between AF and serum urate in Nielsen's study.

Supplemental Figure 16. The scatter plot for the association between AF and serum

urate in Nielsen’s study.

Supplemental Figure 17. The funnel plot for the association between AF and serum urate in Nielsen’s study.
